# Genetic diversity, population structure, and a genome-wide association study of sorghum lines assembled for breeding in Uganda

**DOI:** 10.3389/fpls.2024.1458179

**Published:** 2024-10-07

**Authors:** Faizo Kasule, Boris M. E. Alladassi, Charles John Aru, Scovia Adikini, Moses Biruma, Michael Adrogu Ugen, Ronald Kakeeto, Williams Esuma

**Affiliations:** ^1^ Interdepartmental Genetics and Genomics (IGG), Iowa State University, Ames, IA, United States; ^2^ Department of Agronomy, Iowa State University, Ames, IA, United States; ^3^ National Semi-Arid Resources Research Institute (NaSARRI), Soroti, Uganda; ^4^ National Crops Resources Research Institute (NaCRRI), Kampala, Uganda

**Keywords:** DArT-seq, genetic variation, GWAS, linkage disequilibrium, SNPs, sorghum

## Abstract

Sorghum is an important source of food and feed worldwide. Developing sorghum core germplasm collections improves our understanding of the evolution and exploitation of genetic diversity in breeding programs. Despite its significance, the characterization of the genetic diversity of local germplasm pools and the identification of genomic loci underlying the variation of critical agronomic traits in sorghum remains limited in most African countries, including Uganda. In this study, we evaluated a collection of 543 sorghum accessions actively used in Ugandan breeding program across two cropping seasons at NaSARRI, Uganda, under natural field conditions. Phenotypic data analysis revealed significant (*p*<0.01) variation among accessions for days to 50% flowering, plant height, panicle exsertion, and grain yield, with broad-sense heritability (H²) estimates of 0.54, 0.9, 0.81, and 0.48, respectively, indicating a high genetic variability for these traits. We used a newly developed genomic resource of 7,156 single nucleotide polymorphism (SNP) markers to characterize the genetic diversity and population structure of this collection. On average, the SNP markers exhibited moderately high polymorphic information content (PIC = 0.3) and gene diversity (He = 0.3), while observed heterozygosity (Ho = 0.07) was low, typical for self-pollinating crops like sorghum. Admixture-based models, PCA, and cluster analysis all grouped the accessions into two subpopulations with relatively low genetic differentiation. Genome-wide association study (GWAS) identified candidate genes linked to key agronomic traits using a breeding diversity panel from Uganda. GWAS analysis using three different mixed models identified 12 genomic regions associated with days to flowering, plant height, panicle exsertion, grain yield, and glume coverage. Five core candidate genes were co-localized with these significant SNPs. The SNP markers and candidate genes discovered provide valuable insights into the genetic regulation of key agronomic traits and, upon validation, hold promise for genomics-driven breeding strategies in Uganda.

## 1 Introduction

Sorghum (*Sorghum bicolor* (*L*.) Moench) is a major cereal crop produced worldwide (Smith and Frederiksen, 2000). It is diploid (*2n = 2x =* 20) with an estimated genome size of 735Mbp (Paterson et al., 2004). Sorghum domestication is believed to have started about 3000 to 4000 BC in East Africa (Winchell et al., 2017). As the fifth most produced cereal crop after wheat, rice, maize, and barley (FAO, 2023a), sorghum is a multi-use crop valued for its versatility as a source of food, feed, forage, and biofuels (Salas-Fernandez et al., 2009; FAO, 2023b). The sorghum grains are rich in essential nutrients, including carbohydrates, protein, crude fiber, and minerals such as iron and zinc (Adebo and Kesa, 2023; FAO, 2023b).

The adaptability of sorghum to adverse soil and weather conditions, where other cereals struggle, makes it a crucial crop in drought-prone regions, particularly in sub-Saharan Africa (Andiku et al., 2022; Adedugba et al., 2023). The crop is essential for food security and agricultural sustainability, especially in arid and semi-arid regions characterized by challenging agroclimatic conditions (Andiku et al., 2022; FAO, 2023a).Sorghum covers approximately 40.8 million hectares worldwide, with a global production of 57.9 million metric tons (MMT). Africa has a total area of 29.1 million ha producing 29.6 MMT, with East Africa producing 7.3 MMT from 5.1 million ha (FAO, 2023a). Uganda, the fourth-largest sorghum producer in East Africa, generates around 225,000 tons of grain from approximately 470,083 hectares (FAO, 2023a). Sorghum in Uganda ranks third among cereals, after maize and rice, and is grown for food, brewing, and forage purposes (Andiku et al., 2021; UBOS, 2022). The crop exhibits wide adaptability, thriving in diverse regions from the highlands of Kigezi in Western Uganda to lowland and subhumid areas in East and Northern Uganda (Awori et al., 2015; Mugagga et al., 2020).

However, sorghum production has declined from 457,000 tons in recent years (UBOS, 2022). This decline is attributed to farmers using unimproved varieties, drought, lack of inorganic fertilizer, pests and diseases, high costs of production inputs, bird damage, limited market access, unavailability of inputs, small land holdings, and insufficient agricultural extension services.

These factors have led to low on-farm yields of 0.8 tons/ha, compared to a potential yield of 3-5 t/ha (Awori et al., 2015; Andiku et al., 2021). Sustainable breeding and promotion of improved cultivars are necessary for increasing the on-farm sorghum yields in Uganda. Therefore, there is a need to study the existing genetic diversity to boost local breeding efforts to develop high-yielding and adaptable sorghum varieties in Uganda.

The primary and necessary step of every breeding program is to characterize the genetic diversity existing within the initial germplasm pool for target traits. This is a crucial strategy breeders use to design selection schemes and enhance crop performance (Dillon et al., 2007; Mamo et al., 2023). Researchers globally exploit genetic resources to analyze trait variations for developing superior genotypes with high-yield components, enhanced quality, and resilience to environmental stresses (Morris et al., 2013; Boyles et al., 2016).The predominant approach in Uganda has been to characterize sorghum genetic diversity primarily through morphological traits like days to flowering, plant height, panicle exsertion, grain color, yield and size among others (Akatwijuka et al., 2019; Andiku et al., 2022; Apunyo et al., 2022). However, morphological markers often have limitations, including low polymorphism and heritability, and are influenced by environmental conditions (Mbeyagala et al., 2012; Chakrabarty et al., 2022; Mufumbo et al., 2023).

In the last decade, next-generation sequencing technologies (NGS), especially genotyping by sequencing (GBS), have become prevalent for discovering single nucleotide polymorphisms (SNPs) in sorghum trait mapping and diversity studies (Morris et al., 2013; Boyles et al., 2016; Faye et al., 2021; Enyew et al., 2022; Gimode et al., 2024). Previous studies on sorghum genetic diversity have been limited and often narrow in scope, with most researchers focusing on a few accessions of the entire germplasm found in the Uganda breeding program (Mbeyagala et al., 2012; Akatwijuka et al., 2019; Apunyo et al., 2022). The genetic diversity and population structure of sorghum lines from Africa, Asia, and the USA assembled at the National Semi-Arid Resources Research Institute (NaSARRI) Genebank has remained undocumented.

Genome-wide association studies (GWAS) have successfully identified genomic regions linked to various traits in sorghum across other African countries (Girma et al., 2019; Habyarimana et al., 2020; Enyew et al., 2022; Maina et al., 2022). Previous GWAS have identified numerous genomic regions associated with various agronomic traits for genome-assisted breeding in sorghum (Morris et al., 2013; Boyles et al., 2016; Zhao et al., 2016). In Uganda, GWAS efforts have so far focused exclusively on the sorghum core collection housed in the National Genebank (Chakrabarty et al., 2022; Mufumbo et al., 2023). However, there has been no GWAS on the sorghum breeding lines actively used by the National Agricultural Research Organization (NARO), which leads sorghum breeding efforts in Uganda. Therefore, the use of GWAS on germplasm at NaSARRI Genebank holds significant potential in unveiling genomic regions associated with targeted traits, leading to gene discovery for important traits for the Ugandan sorghum breeding program.

The objective of this study was to assess the genetic diversity and population structure of a mini-core collection of 543 accessions at the NaSARRI Genebank in Uganda using GBS-derived SNP markers. Additionally, this study aimed to identify genomic loci and corresponding candidate genes associated with key agronomic traits such as plant height, days to 50% flowering, panicle exsertion, glume coverage, and grain yield through GWAS. Together, these findings provide insights into the genetic basis of important traits to facilitate sorghum improvement, conservation, and utilization in Uganda.

## 2 Materials and methods

### 2.1 Plant materials

**Figure 1:**
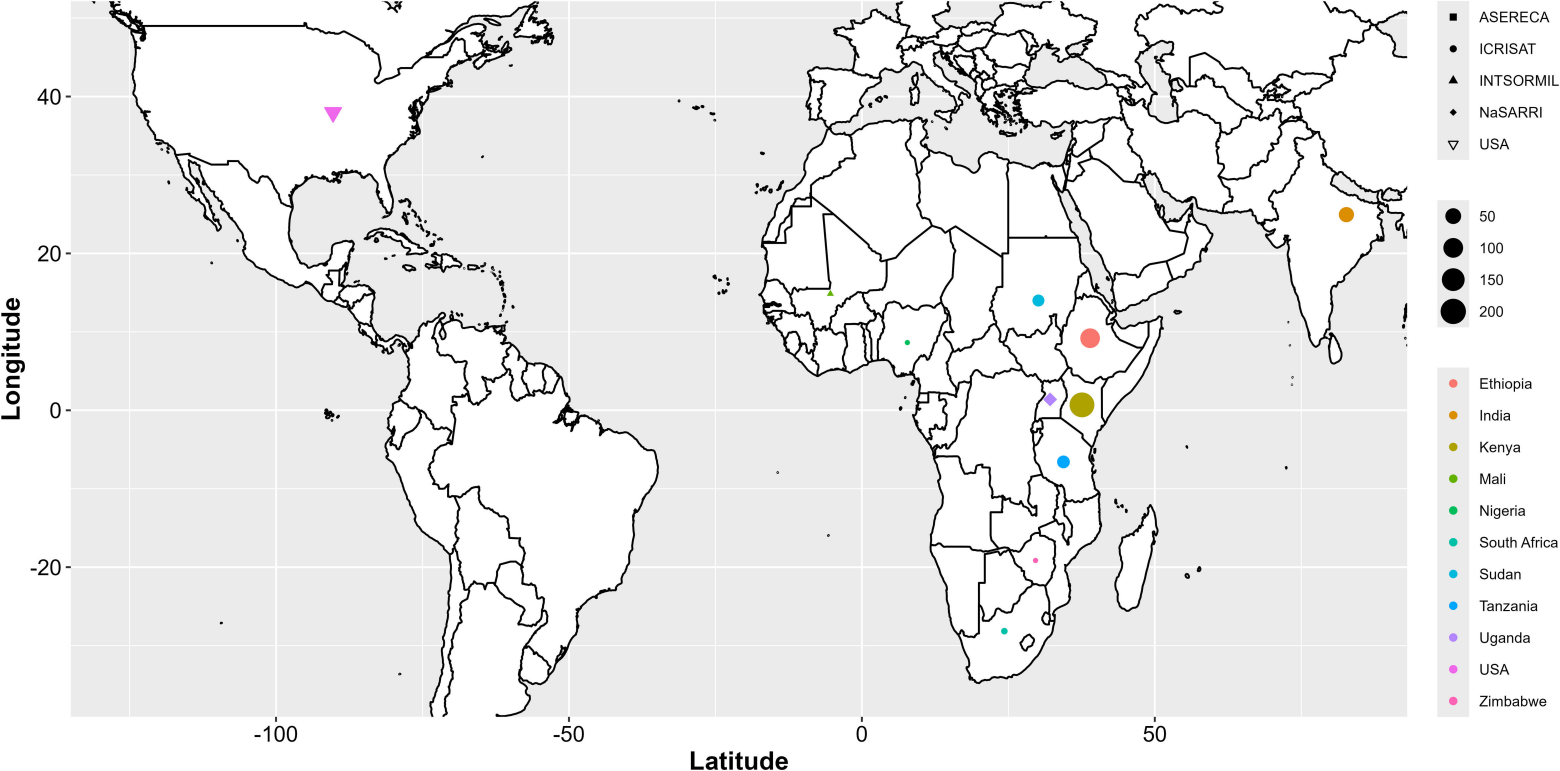
Geographical origin of 543 sorghum accessions used in the study.

The mini-core collection of sorghum used in this study consisted of 543 sorghum accessions, representing approximately 90% of the available non-segregating germplasm for breeding, sourced from 11 countries (**Supplementary Table S1**). The sorghum seeds were obtained from the National Semi-Arid Resources Research Institute (NaSARRI) Genebank of Uganda. These accessions originated from the International Crops Research Institute for the Semi-Arid Tropics (ICRISAT; 462), NaSARRI (43), USA (Purdue University; 30), the Association for Strengthening Agricultural Research in Eastern and Central Africa (ASERECA; 4), and the International Sorghum and Millet Collaborative Research Support Program (INTSORMIL CRSP; 4) (**Figure 1**).

### 2.2 Field trials and phenotyping

The mini-core collection was evaluated under natural field conditions at NaSARRI (1°39’N and 33°27’E; 1140 m above sea level; average rainfall 1427mm per year; average temp 24°C; sandy loam soils) for two consecutive cropping seasons; the second rainy season of 2021 and the first rainy season of 2022. Sorghum was planted in different fields whose previous crop was greengram (*Vigna radiata* (L.) R. Wilczek var. *radiata*) and groundnuts (*Arachis hypogaea* L.) for 2021 and 2022, respectively. The field experiment was laid out in a 10 x 60 augmented block design comprising four checks: EPURIPUR, NAROSORG 3, SESO 1, and SESO 3. Each block randomly allotted the checks, with plot sizes of 1.8 m x 2 m. To address the lack of full randomization across replications, we used the Breeding Management System (BMS) of the Integrated Breeding Platform (IBP) available at https://www.integratedbreeding.net to randomly assign experimental units, with separate randomization applied for each cropping season. The experimental units in both seasons were maintained following Uganda’s standard agronomic practices of sorghum (Andiku et al., 2022). Phenotypic data was collected on 10 randomly selected and tagged plants per plot following the descriptors of sorghum (IBPGR, 1993). Quantitative traits included days to 50% flowering (DTF, days), plant height (PH, cm), panicle exsertion (PE, cm), and grain yield (GY, kg/ha). DTF was recorded when half of the panicles and 50% of plants had attained anthesis within a plot. PH was measured from the ground level to the tip of the panicle at physiological maturity by randomly selecting 10 plants in a test plot. PE was the length of the peduncle from the flag leaf to the base of the inflorescence and was measured by randomly sampling 10 plants in a test plot at full maturity. GY was the grain weight per plot (kg) at 12.5% moisture content was recorded in terms of weight per unit area (kg/ha). The grain was harvested manually per plot. Glume coverage (GC); amount of grain covered by glume was scored based on the following scale 1 = 25%, 2 = 50%, 3 = 75%, 4 = 100% or grain fully covered, and 5 = glumes longer than the grain.

### 2.3 Genotyping

Genotyping was performed using medium-density DArTseq technology as described by Kilian et al. (2012). Briefly, leaf tissues were sampled from young leaves three weeks after planting from a single plant in a test plot growing under natural field conditions at NaSARRI during the 2022 cropping season. Four leaf discs of 6 mm diameter were punched into wells of sample collection plates and desiccated using silica gel for 48 hours. Plates containing dry leaf tissues were shipped to SEQART AFRICA (https://www.seqart.net/) located at the Biosciences Eastern and Central Africa (BecA-ILRI) Hub in Nairobi for DArTseq genotyping (Elshire et al., 2011). DNA was extracted using the Nucleomag Plant Kit, yielding 50-100 ng/µl of genomic DNA, which was quality-checked on 0.8% agarose gels. The DNA was digested with *PstI* and *HpaII* restriction enzymes, and libraries were prepared for single-read sequencing on the Illumina HiSeq2500 platform, achieving a depth of 1.2 million reads per sample. Marker scoring was performed using DArTsoft14, producing binary (presence/absence) SilicoDArT and SNP markers. Sequencing reads were aligned to the sorghum reference genome BTx63 *v*3 obtained from Phytozome (phytozome-next.jgi.doe.gov). Imputation was performed using the probabilistic principal component analysis (PPCA) method, as described by Stacklies et al. (2007). PPCA was chosen because it demonstrated the highest simple matching coefficient of 84.21% among the five methods tested. Phenotypic data analysis.

The phenotypic data for the two years (environments) was initially analyzed separately using the *Augmented RCBD* function from the *agricolae* package in R. Subsequently, a combined analysis was performed using a restricted maximum likelihood (REML) linear mixed effects model with the *LmerTest* package in R. The following linear model was fitted for the combined analysis:


$$y_{ijkl}=\;E_{i}+\;C_{j}+\;G:Switch_{k}\;+\;E*C_{ij}+\;E*{\left(G:Switch\right)}_{ik}+\;e_{ijkl}$$

Where $y_{ijkl}$ is the individual observation made in year i; $E_{i}$ is the effect of year i, random effects; $C_{j}$ is the effect of check (inbred parent) j, fixed effects, $G:Switch_{k}$ is the effect of genotype k, random effects, Switch is a dummy variable, 0 for check and 1 for testing genotype, $E*C_{ij}$ is the effect of the interaction between year i and check j, random effects, $E*{\left(G:Switch\right)}_{ik}$ is the effect of the interaction of year i and testing genotype k, random effects, and $e_{ijkl}$ is the random error associated with the observation $y_{ijkl}$ .

The broad-sense heritability was computed for combined analysis as follows:


$$\frac{\sigma_{G}^{2}}{\sigma_{G}^{2}+\frac{\sigma_{GXE}^{2}}{n}+\frac{\sigma_{e}^{2}}{n}}$$

where, $\sigma_{G}^{2}$ is the genetic variance, $\sigma_{GXE}^{2}$ is the variance of the genotype by environment interaction, $\sigma_{e}^{2}$ as the residual variance, and n is the number of years (environments).

Correlation analysis was performed using the *ggpairs* function from the *GGally* R package.

### 2.4 Data quality control, filtering, SNP imputation

The imputed marker data provided by Diversity Arrays Technology (DArT) Seq in the single row format was imported in R using the *gl.read.dart* function from *dartR* package. The SNPs that were monomorphic, with 20% or more missing data, or had a minor allele frequency (MAF) below 5% were filtered out using the inbuilt functions of the *dartR* package. Also, markers with undetermined alignment to the reference genome were removed. From a total of 20,211 raw DArTSeq single nucleotide polymorphism (SNP) markers, were narrowed to 7,156 high confidence SNP markers (35.4%) after filtering spread over the 10 chromosomes of *Sorghum bicolor.* Genetic parameters such as minor allele frequency (MAF), expected heterozygosity (He), observed heterozygosity (Ho), and polymorphism information content (PIC) for each marker were calculated using inbuilt functions of *dartR* R package to determine the degree of variation among the SNP markers.

### 2.5 Population structure analysis

The population structure of the sorghum mini-core collection was assessed using filtered data of 7,156 SNP markers. Three methods were employed and compared to infer the population structure of the panel. First, the STRUCTURE program, version 2.3.4 (Pritchard and Rosenberg, 1999; Pritchard et al., 2000), was run 10 times for each assumed number of subpopulations (K = 1 to 11), using the admixture model with the main parameters set at 20,000 for burn‐in and 20,000 MCMC replicates after burn-in. For each value of K, a bar plot of the best run having the highest likelihood value was created. The delta K plot indicated a peak at K = 2, which, following Evanno et al. (2005), was selected as the most likely number of subpopulations.The results obtained from STRUCTURE were analyzed in Structure Harvester software (Earl and vonHoldt, 2012), to infer the optimum number of subpopulations based on deltaK metrics. Second, principal component analysis (PCA) was performed in R using the *prcomp* function in the *stats* package. The PC scores of the sorghum accessions on the first three axes were plotted as a biplot using *ggplot2* R package.

The third approach consisted of cluster analysis using the neighboring-joining tree estimation. The data was analyzed using the *adegenet* R package to compute pairwise frequency-based distance among accessions using the CSChord distance measure (Cavalli-Sforza and Edwards, 1967). Using the *ape* package, the distance matrix obtained from *adegenet* was used to construct a Neighbor-joining tree in R (Paradis et al., 2004). To assess the concordance among the three methods, the biplot of the PCA and the Neighbor-joining tree of the cluster analysis were plotted by color-coding the sorghum accessions based on their subpopulations inferred by structure analysis Lastly, to estimate the components of variance among and within populations, an analysis of molecular variance (AMOVA) was performed as described by Excoffier et al. (1992) and implemented in the *ade4* R package. To estimate population differentiation, the fixation index (F_ST_) of the two populations was estimated as the ratio of the variance between populations to the pooled variance within populations obtained from the AMOVA.

### 2.6 Linkage disequilibrium analysis

Pairwise linkage disequilibrium (LD), measured as the squared correlation of allele frequencies, *r^2^*, was estimated for all 7,156 SNP markers based on their physical distance using the *LD.decay* function from the R package *sommer* (Covarrubias-Pazaran, 2016). The LD decay curve was fitted using smoothing spline regression described by Hill and Weir (1988), to fit the genome-wide LD decay curve line. The *r^2^* values and the LD decay curve line were plotted against the physical distance between each pair of markers using the R package *ggplot2*.

### 2.7 Genome-wide association studies

Genome-wide association studies (GWAS) were conducted to identify genomic loci associated with major agronomic traits using best linear unbiased estimations (BLUEs) of the accessions with data obtained from both the individual season and combined analyses. The results of three models used and compared; the unified mixed linear model (MLM; Yu et al., 2006), the multi-locus mixed model (MLMM; Segura et al., 2012), and the fixed and random model circulating probability unification (FarmCPU; Liu et al., 2016) as implemented in the R package *GAPIT* v.3 (Wang and Zhang, 2021). All three models minimize the false discovery rate by accounting for both population structure and kinship. Further, the Benjamini-Hochberg procedure was used to determine the adjusted genome-wise 5% significance threshold accounting for multiple testing. Marker-trait association with statistical significance for at least two environments or two GWAS models were highlighted on the Manhattan plot using the *CMplot* function from the R package *rMVP* (Yin et al., 2021). Additionally, Q-Q plots were generated using the same *CMplot* function within the *rMVP* package. Candidate genes were identified using the sorghum reference genome *BTx623 (v3)* in the SorghumBase database (Gladman et al., 2022). Annotated genes located within 100 kbp of the physical positions of each significant SNP were considered as candidate genes. Biological processes and molecular function of the candidate gene were reported. Additionally, putative plant organs for candidate gene expression were obtained from their existing expression atlas within SorghumBase and Gramene database.

## 3 Results

### 3.1 Phenotypic variations and heritability of agronomic traits

A combined analysis of variance across seasons showed significant differences (*p*< 0.001) among genotypes and years for all agronomic traits (**Table 1**). The ANOVA indicated substantial genetic variability among genotypes for days to 50% flowering (DTF), plant height (PH), panicle exsertion (PE), and grain yield (GY) (*p* ≤ 0.001). Year effects were highly significant for all traits, while genotype-by-year interactions were only significant for PH (*p* ≤ 0.001) (**Table 1**). There was a substantial phenotypic variation among the sorghum accessions for all agronomic traits (**Supplementary Table S2**). Days to 50% flowering (DTF) ranged from 58 to 117 days after planting with a mean of 83 days, while plant height (PH) ranged from 67 to 319.5 cm, with a mean of 142.7 cm. The panicle exsertion (PE) of the accessions ranged from 0 to 25.5 cm with a mean of 3 cm. The average grain yield (GY) was 1100 kg/ha, while individual accessions produced 140 to 8300kg/ha.

**Table 1:** Analysis of variance for four quantitative traits in sorghum germplasm assessed across two seasons (year) at NaSARRI in Uganda.

SOV	Df	DTF	PH	PE	GY
Year	1	73790.00***	46168.00***	265.76***	16921875.00***
Block (Year)	18	22.30^ns^	163.60ns	8.16^ns^	321663.00*
Checks	3	549.80^ns^	10018.40***	62.72^ns^	1431970.00^ns^
Genotypes	561	62.00***	2350.00***	33.24***	388100.00***
Year x Checks	3	204.70***	28.70^ns^	11.55^ns^	401556.00^ns^
Year x Genotypes	557	28.00^ns^	234.00***	6.31^ns^	200313.00^ns^
Residuals	54	28.80	115.20	6.35	144980.00

Df, Degree of freedom; DTF, Days to 50% flowering (days); PH, Plant height (cm); PE, Panicle exsertion (cm); GY, Grain Yield (kg/ha); ^ns^p> 0.05, ^∗∗∗^p ≤ 0.001.

**Figure 2:**
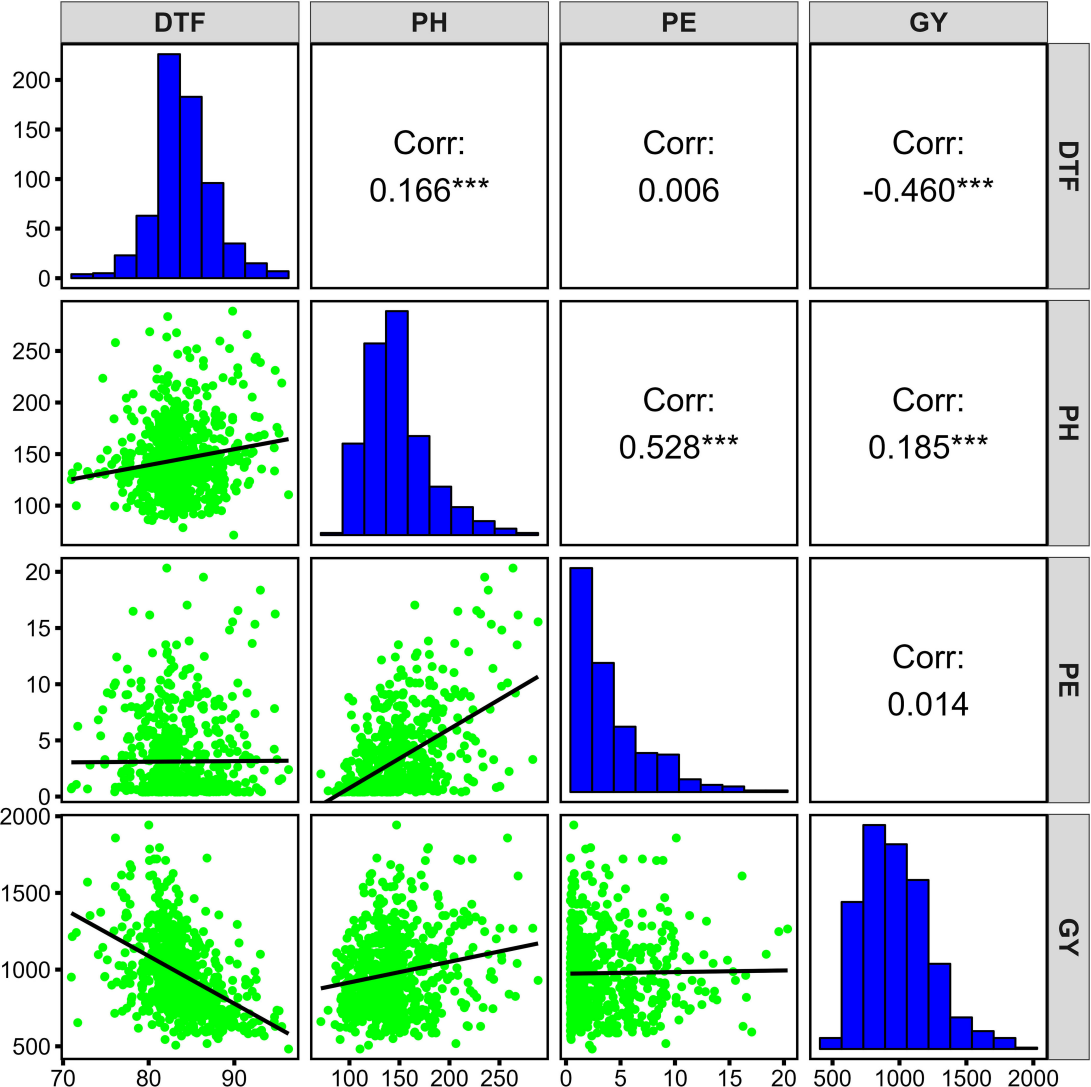
Phenotypic distribution and correlations of four agronomic traits in the diversity panel. *** *p* ≤ 0.001.

The broad-sense of heritability (*H^2^*) was high for PH (0.9), and PE (0.81), whereas it was moderate for DTF (0.54) and GY (0.48) (**Supplementary Table S2**).There was a low to moderate positive correlation between PH and PE (*r =* 0.528, *p*<0.001), GY (*r* = 0.185, *p*<0.001), and DTF (*r* = 0.166, *p*<0.001) (**Figure 2**). There was a significant negative correlation between DTF and GY (*r* = -0.46, p<0.001). No significant association was observed between PE and DTF (*r* = 0.006) and GY (*r* = 0.014).

### 3.2 Genomic marker density and genetic diversity

**Figure 3:**
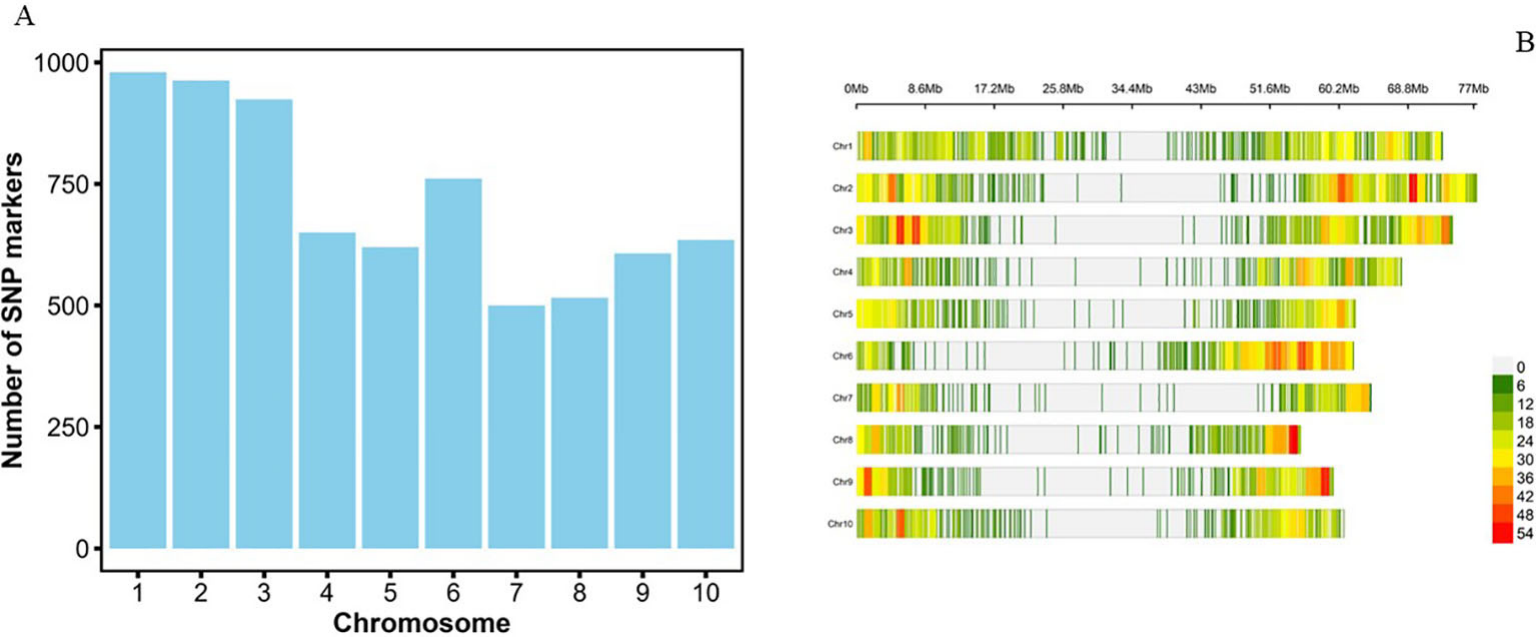
**(A)** Distribution of 7,156 SNPs across 10 sorghum chromosomes. **(B)** Distribution of SNPs within the 1Mb window size across the 10 chromosomes of sorghum.

The largest number of SNPs was identified on chromosome 1 (980 SNPs; 13.7%) followed by chromosomes 2 (963; 13.5%) and 3 (924; 12.9%), while chromosomes 8 (516; 7.2%) and 7 (500; 7.0%) had the least SNPs (**Figure 3A**). The density of SNP markers was plotted per 1 Mb window across all chromosomes. The size of the chromosomes ranged from 55 to 77 Mb, with an average of 65.4 SNPs per Mb of the genome. The marker density ranged from 1 to 55 SNPs per Mb across the 10 chromosomes (**Figure 3B**; **Supplementary Figure S1**). The highest SNP density of 55 SNPs per Mb was observed on chromosome Chr8, while all ten chromosomes had a region with a density of 1 SNP per Mb. As expected, there was a general trend of low density of markers around the centromeric regions of all the chromosomes (**Figure 3B**). The summary statistics of the 7,156 SNP markers are presented in **Table 2**. The mini-core collection exhibited important diversity, with minor allele frequency (MAF) of the final set of the markers ranging from 0.05 to 0.5 (Mean = 0.21), while the polymorphism information content (PIC) ranged from 0.09 to 0.5 (Mean = 0.3). The expected heterozygosity (He) ranged from 0.1 to 0.5 with a mean value of 0.3, while the observed heterozygosity (Ho) varied from 0 to 0.8 with a mean of 0.07 (**Table 2**).

**Table 2:** Summary statistics of diversity indices of 543 sorghum accessions based on 7,156 SNP markers.

Genetic Parameters	Statistics			
	Mean	Median	Min	Max
Minor allele frequency (MAF)	0.21	0.18	0.05	0.5
Observed heterozygosity (Ho)	0.07	0.05	0	0.8
Expected heterozygosity (He)	0.3	0.3	0.1	0.5
Polymorphism information content (PIC)	0.3	0.29	0.09	0.5

Min, Minimum; Max, Maximum.

### 3.3 Inferring population structure

**Figure 4:**
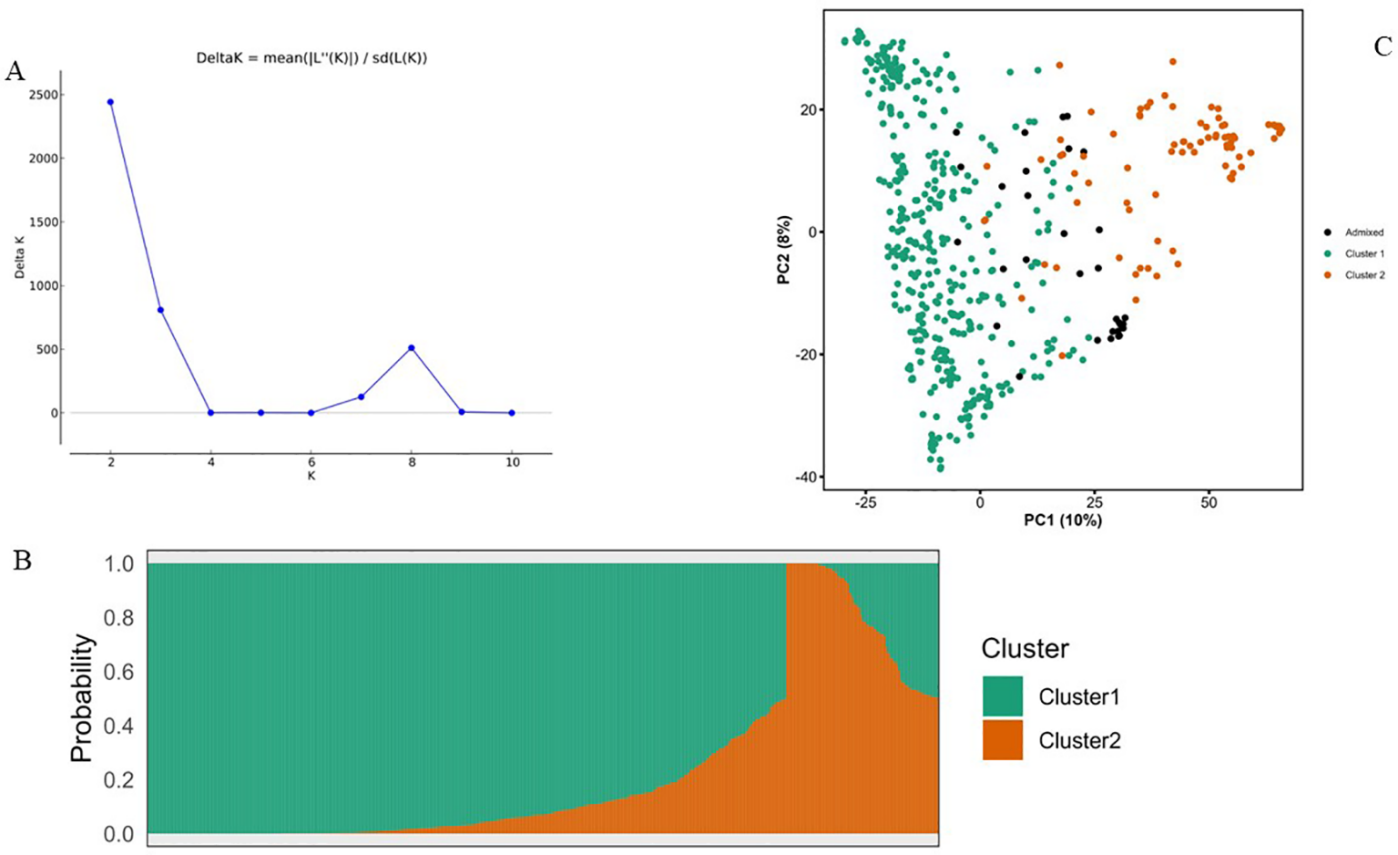
**(A)** A graph of estimated membership fraction based on Structure Analysis. The maximum Δk determined by the Structure harvester was K= 2, indicating the entire population could be grouped into two clusters. **(B)**The Structure Plot for K = 2 at individual and across iteration of 543 sorghum accessions. **(C)** Principal component analysis (PCA) plots showing 543 sorghum accessions into two subpopulations based on 7,156 SNP markers. PC1 and PC2 are the first, and second principal components, respectively.

To infer the population structure of the NaSARRI sorghum mini-core collection, we performed an admixture-based analysis on the 7,156 SNPs. The delta K statistic peaked at K= 2, (**Figure 4A**). This indicates that the population structure revealed the presence of two genetic subpopulations within the mini-core collection of 543 sorghum accessions used in this study (**Figure 4B**). Based on the likelihood values of inferred ancestry, a total of 427 accessions (78.64%) were assigned to subpopulation 1, while 82 accessions (15.10%) were assigned to subpopulation 2, and 34 accessions (6.26%) were admixed with alleles inherited from both genetic subpopulations (**Figure 4B**). In subpopulation 1, most of the accessions originated from ICRISAT (356), followed by NaSARRI (40), while in Cluster 2, most accessions originated from ICRISAT (75), followed by USA (4), and the admixed category comprised of 31 accessions with a mix of alleles from different genetic background (**Table 3**).

**Table 3:** Cluster-wise distribution of 543 sorghum accessions assembled in Uganda by origin using 7,156 DArT SNP markers.

Inferred membership	ASERECA	ICRISAT	INTSORMIL	NaSARRI	USA	Total
Cluster 1	3	356	3	40	25	427
Cluster 2	0	75	1	2	4	82
Admixed	1	31	0	1	1	34
Total	4	462	4	43	30	543

The results from the principal component analysis (PCA) showed that the first two principal component axes explained 18% of the genetic variance among the SNP markers (**Supplementary Figure S2**). The clustering pattern depicted by the PCA biplot agreed with the population structured revealed by the analysis from STRUCTURE and further confirmed that the 543 sorghum accessions from this study were grouped into two clusters, and a few were grouped within a different population forming admixtures (**Figure 4C**).

**Figure 5:**
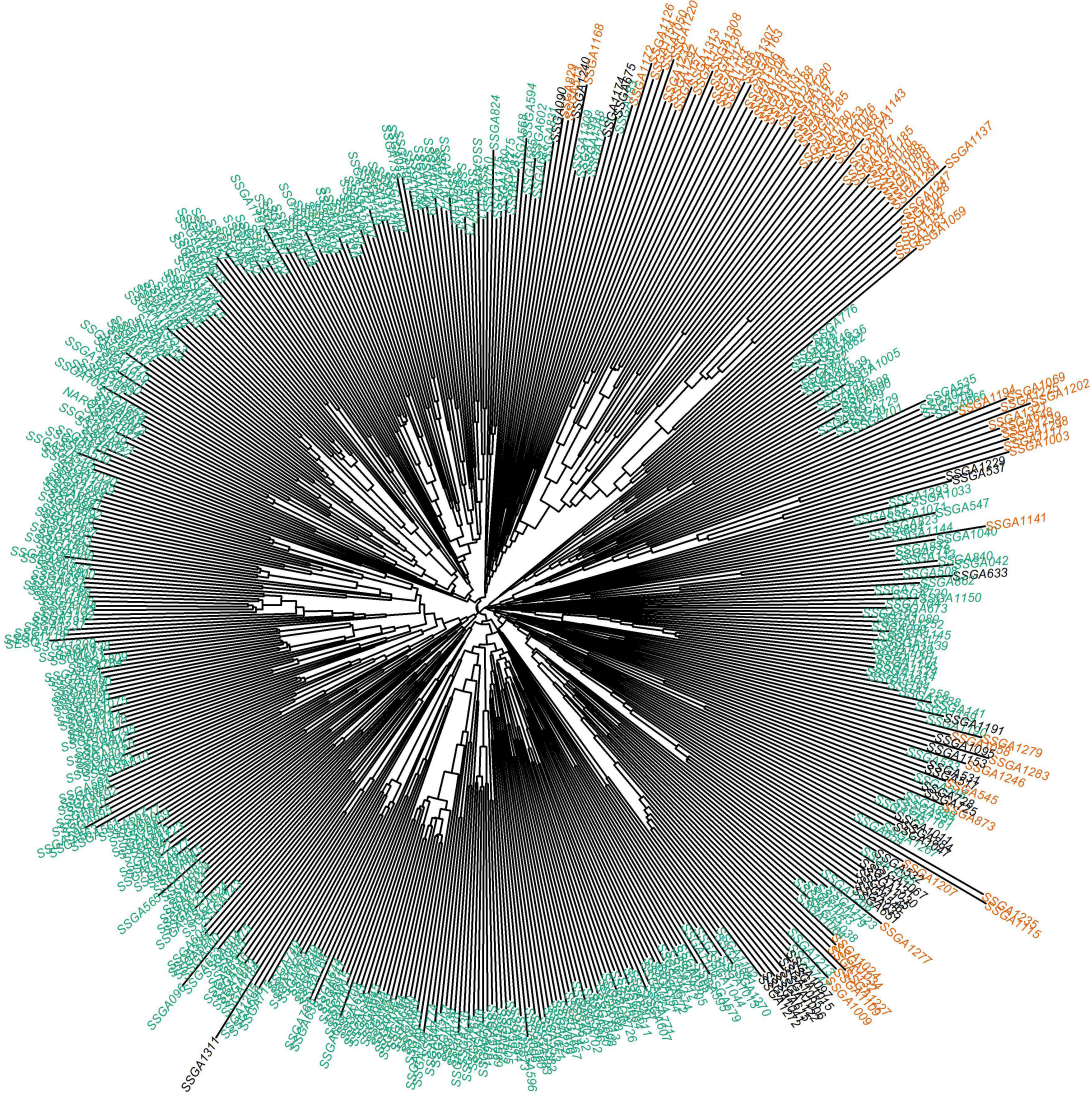
Phylogenetic analyses of 543 sorghum accessions using the neighbor-joining method. Different colors depict the structure analysis generated populations. Colors represent different subpopulations of the germplasm; Green color = subpopulation1, orange = subpopulation2.

The genetic distance among the population was represented by a neighbor-joining dendrogram (**Figure 5**). The dendrogram was constructed for 543 sorghum accessions and color-coded based on the inferred ancestry from STRUCTURE analysis. Overall, the cluster analysis grouped the accessions into two clusters in concordance with the STRUCTURE (**Figure 4B**) and principal component analysis (**Figure 4C**). However, about 20% of the accessions showed admixture of the two subpopulations inferred from STRUCTURE.

Given the concordance observed among all three methods of population structure analysis, the two populations inferred by STRUCTURE were used to perform an analysis of molecular variance (AMOVA) and calculate the pairwise fixation index (F_ST_) (**Table 4**). The AMOVA revealed that 11.9% of the total genetic variance was due to the differentiation between the two subpopulations, while the majority (88.1%) was due to the variation observed within subpopulations. As a result, the estimate of allele frequency differentiation (F_ST_) between the two subpopulations was 0.12.

**Table 4:** Analysis of molecular variance among and within two subpopulations of 543 sorghum accessions evaluated based on 7,156 SNP markers.

Source of variation	df	Sum square	Mean square	Variance component	Proportion of Variance (%)	F_ST_
Subpopulations	1	164833	164833	472.81	11.9	0.12
Accessions within subpopulations	541	1892160	3497.52	3497.52	88.1	
Total	542	2056993				

Df, degrees of freedom; F_ST_, allele frequency differentiation.

### 3.4 Linkage disequilibrium

**Figure 6:**
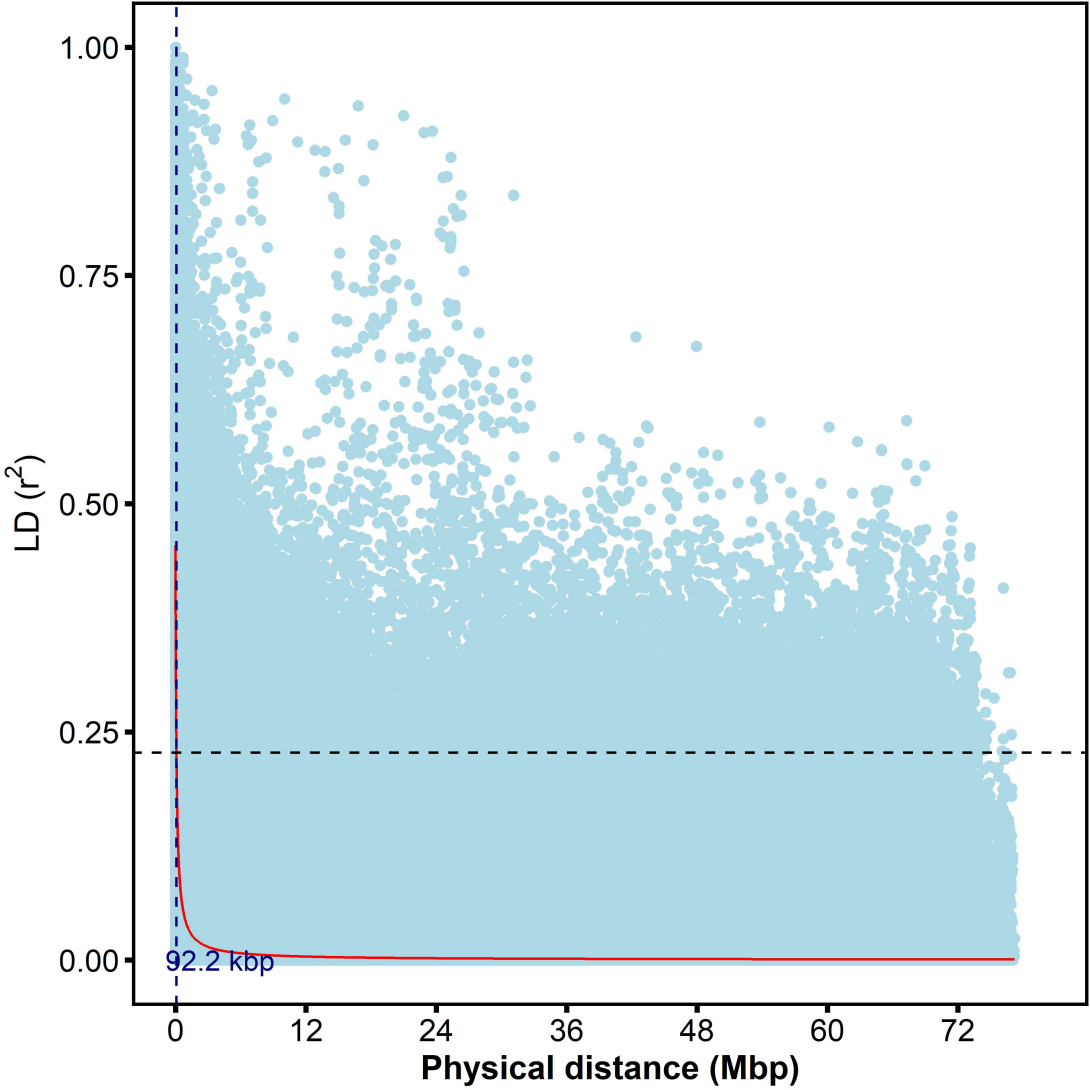
The scatter plot of genome-wide linkage disequilibrium (LD) decay was determined based on the r^2^ values of the marker pairs. The horizontal dotted black line is the half decay r^2^ value of the genome (r^2^=0.23) whereas the vertical blue line is the genetic distance between markers (92.2kbp) at the intersection between the half decay and the LD decay curve.

The ten average pairwise estimate of linkage disequilibrium for the ten chromosomes was similar, with *r^2^* values ranging from 0.067-0.077 and an overall average of 0.070 (**Supplementary Figure S3**). However, notable differences were observed among chromosomes, with chromosome 8 having the highest average *r^2^* (0.077) and Chromosome 6 having the lowest (0.067) among significant marker pairs. The highest and lowest number of significant marker pairs were recorded on Chromosome 1 (254,769) and Chromosome 7 (68,482), respectively (**Supplementary Table S3**). At the genome level, the *r^2^* value was (0.07), and the decay curve of the LD began at *r^2^* value of (0.45) (**Supplementary Figure S3**) and reached half-decay at 0.23 (**Figure 6**). The decay curve of the LD intersected the half-decay line at a distance of 92.2 kb (**Figure 6**). Generally, there was a rapid LD decay with increasing physical distance along the 10 sorghum chromosomes.

### 3.5 Marker-trait association analyses

**Figure 7:**
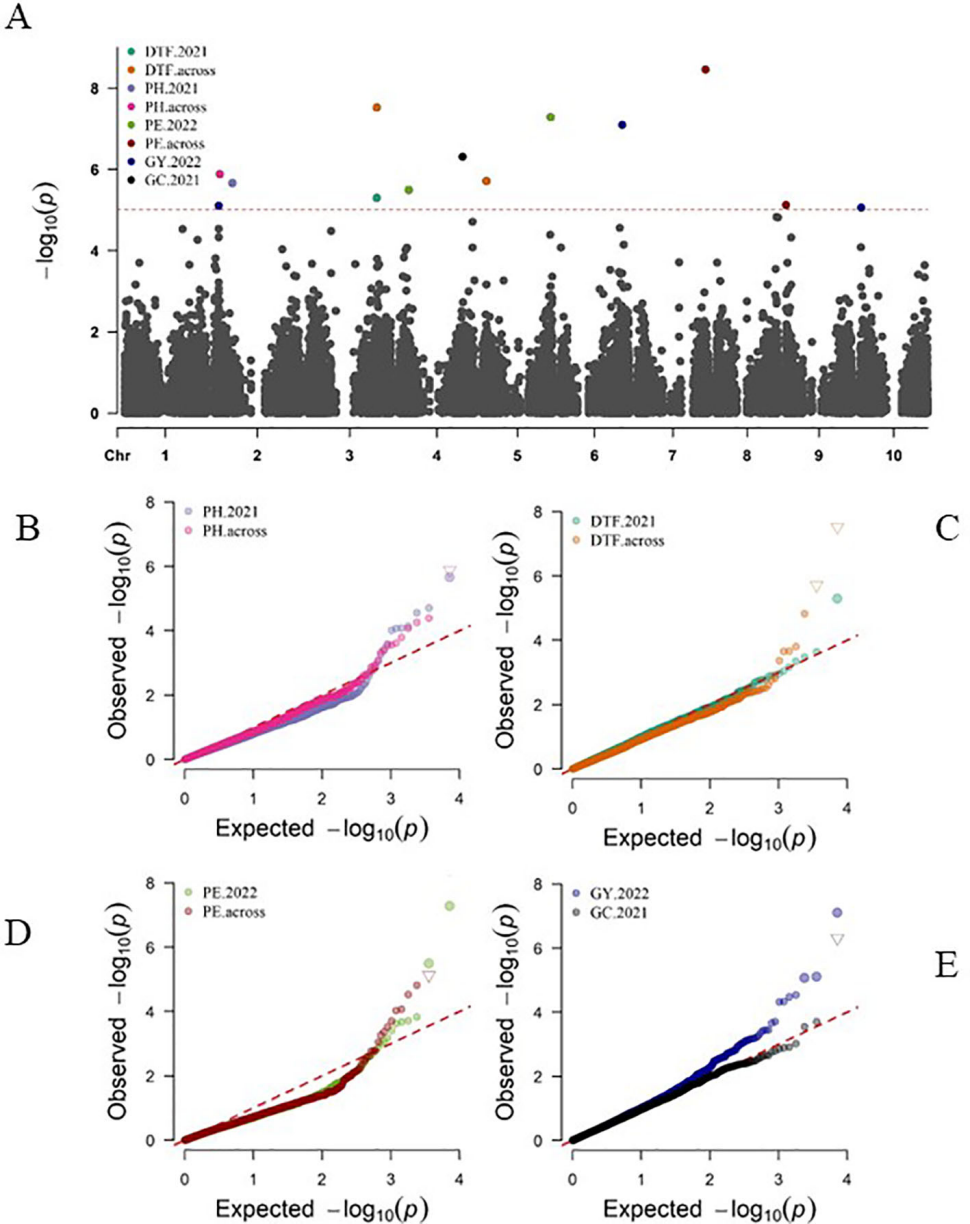
**(A)** Manhattan plot and Q-Q plots **(B–E)** depicting significant marker-trait associations for five agronomic traits using 7,156 genome-wide SNP markers of 543 sorghum accessions with genome- wise Bonferroni adjusted *p*-value of 7e-6 corresponding to -log10(p-value) threshold of 5.16. The Q-Q plots of expected versus observed significance levels for **(B)**, PH, plant height, **(C)** DTF, days to 50% flowering, **(D)** PE, panicle exsertion, **(E)** GC, glume coverage and GY, grain yield.

Genome-wide association studies were performed for five phenotypic traits: PH, DTF, PE, GC, and GY using three different models. In total, the GWAS revealed 13 SNPs associated with the variations in the agronomic traits (**Figure 7**; **Supplementary Table S4**). Two quantitative trait nucleotides (QTNs), S2_16670214 and S2_5378133 on chromosome 2, were associated with plant height. Both QTNs were detected by the FarmCPU model. Two QTNs were associated with days to 50% flowering (DTF). The QTN S3_61344759 on chromosome 3 was detected by all three GWAS models (FarmCPU, MLM, MLMM). The second QTN— S5_3569592, located on chromosome 5 at position 3569592— was detected by MLMM. The FarmCPU GWAS model exclusively identified Four QTNs associated with panicle exsertion (PE) (**Figure 7**; **Supplementary Table S4**). From a singular season of 2022, S4_8921335 on chromosome 4 and S5_60770709 on chromosome 5 showed significant associations with PE. For the combined data analysis, S7_61349278 on chromosome 7 and S9_375005 on chromosome 9 were also identified as QTNs associated with variation in PE. One QTN, S4_56863263, on chromosome 4 was associated with glume coverage. This QTN was detected by only the MLMM GWAS model. All three GWAS models detected three QTNs associated with grain yield (GY) (**Figure 7**; **Supplementary Table S4**). S2_4351947 on chromosome 2 displayed a consistently significant association across FarmCPU and MLM, while S6_55680307 on chromosome 6 showed a significant association across FarmCPU, MLM, and MLMM. Similarly, S10_1446937 on chromosome 10 significantly correlated with grain yield across the three GWAS models.

The significant SNPs associated with PH, DTF, PE, GC, and GY were used to identify the putative candidate genes using the sorghum reference genome *BTx623 (v3)* in the SorghumBase database (Gladman et al., 2022) and are presented in **Table 5**. Regarding plant height (PH), S2_5378133 on Chromosome 2 is located within coding sequences of the candidate gene SORBI_3002G056000, which is involved in chromatin binding. Additionally, S2_16670214, also on Chromosome 2 and associated with PH, correlates with SORBI_3002G124600, a gene implicated in nucleic acid binding (**Table 5**). For days to 50% flowering (DTF), the QTN S5_3569592 on Chromosome 5 is intragenic with in the candidate gene SORBI_3005G039000. This gene is associated with carbonate dehydratase activity and zinc ion binding (**Table 5**). For panicle exsertion (PE): S4_8921335 on chromosome 4 is intragenic within candidate gene SORBI_3004G099700, involved in protein phosphorylation and potential regulation of physiological processes. Additionally, S9_375005 on chromosome 9 aligns with SORBI_3009G004200, associated with defense response and salicylic acid-mediated signaling pathways, impacting PE. For glume coverage (GC), S4_56863263 on Chromosome 4 is located within coding sequences of the candidate gene SORBI_3004G219100, which plays a role in GPI anchor biosynthetic processes crucial for membrane protein attachment. For grain yield (GY), S6_55680307 on chromosome 6 and S10_1446937 on chromosome 10 are located within the coding sequences of candidate genes SORBI_3006G207100 and SORBI_3010G01770, respectively. Further research is needed to understand the biological functions of these genes.

**Table 5:** Candidate genes for five agronomic traits.

SNP	Chr	BP	Trait	Candidate genes	Biological function	Molecular function	Developmental expression atlas	Reference study
S2_5378133	2	5378133 (5.378 Mbp)	PH	SORBI_3002G056000 (position: 5.376 – 5.386 Mbp, 70 kpb long, 1544 amino acids)	–	Chromatin binding	Expression in whole stem at seedling stage and in the middle internode and the internode below the peduncle.	(Makita et al., 2015; McCormick et al., 2018)
S2_16670214	2	16670214 (16.670 Mbp)	PH	SORBI_3002G124600 (location: 16.665-16.674 Mbp, 2.7 – 8.1 kbp, 800 amino acids)		Nucleic acid binding	Expression in whole stem	(Makita et al., 2015)
							Four last internodes (top)	(Kebrom et al., 2017)
S5_3569592	5	3569592 (3.570 Mbp)	DTF	SORBI_3005G039000 (3.566 -3.567 Mbp, 1.2 kpb, 278 amino acids)		carbonate dehydratase activity,	Anther, Flower, Pollen, Spikelet, Lower panicle,	(Davidson et al., 2012)
						zinc ion binding		(Makita et al., 2015; Wang et al., 2018)
S4_8921335	4	8921335 (8.921 Mbp)	PE	SORBI_3004G099700(8.916 -8.920 Mbp, 3.8 kbp, 1037 amino acids)	protein phosphorylation	Protein binding, ATP binding	Flag leaf sheath, first leaf emerge, internode below peduncle	(McCormick et al., 2018)
S7_61349278	7	61349278 (61.35 Mbp)	PE	SORBI_3007G180200 (61.366 -61.370 Mbp, 2 kbp, 300 amino acids)			Early inflorescence, pistil,	(Davidson et al., 2012)
							Higher internode	(McCormick et al., 2018)
S9_375005	9	375005 (0.375 Mbp)	PE	SORBI_3009G004200 (0.374 -0.378 Mbp, 2.7kbp, 607 amino acids)	Defense response, regulation of salicylic acid mediated signaling pathway		Early inflorescence, Vegetative meristem	(Davidson et al., 2012)
							Panicle, internode below peduncle	(McCormick et al., 2018)
S4_56863263	4	56863263 (56.86 Mbp)	GC	SORBI_3004G219100 (56.862-56.865 Mbp, 3.2 kbp, 302 amino acids)	GPI anchors the biosynthetic process, membrane of cells, pollen tube growth, pollen germination,		Emerging inflorescence, inflorescence	(Davidson et al., 2012)
S6_55680307	6	55680307 (55.68 Mbp)	GY	SORBI_3006G207100 (55.685-55,686 Mpb, 1.12 kbp, 74 amino acids)			Seed, seed 5 days after pollination, endosperm, inflorescence, pericarp	(Davidson et al., 2012)
S10_1446937	10	1446937 (1.45 Mbp)	GY	SORBI_3010G017700 (1.447-1.449 Mbp, 1.5 kbps, 370 amino acids)			Pollen at booting stage, spikelet, panicle	(Makita et al., 2015; Wang et al., 2018)

SNP, Single nucleotide polymorphism; Chr, chromosome; BP, base pairs; PH, plant height; DF, days to 50% flowering; PE, panicle exsertion; GC, glume coverage; GY, grain yield.

## 4 Discussion

### 4.1 Phenotypic variation

Sorghum plays a vital role in ensuring global food security, serving as a versatile staple food, a bioenergy source, feed for livestock, and a basis for various industrial products (Habyarimana et al., 2020; FAOSTAT, 2023). This study addresses the gap in genetic and genomic research within the sorghum breeding program in Uganda, which has largely depended on phenotypic characterization. The rarity of such genomic studies limits a comprehensive understanding of the genetic diversity within the assembled breeding lines, thus hindering efforts for crop improvement (Chakrabarty et al., 2022; Mufumbo et al., 2023). This study examined the genetic diversity of sorghum breeding accessions using SNP markers for effective management, genetic improvement, and conservation in Uganda. Furthermore, it contributes to advancing high-resolution sequencing methods by laying the foundation for genomics-assisted breeding and conservation of sorghum in the country through fast-tracking population advancement, cultivar development, and varietal release (Girma et al., 2019; Faye et al., 2021; Baloch et al., 2023).

We observed high phenotypic variation among the sorghum accessions constituting the mini-core collection assembled for breeding in Uganda that was used as a GWAS panel for studied traits. The analysis showed significant mean squares attributable to the season (year) for all traits, indicating that the two seasons provided sufficient differentiation of genotypes. The contribution of genotype-by-environment (G × E) interactions was significant but less influential than the genotypic effects, as evidenced by the larger sum of squares for genotypes compared to G × E interaction. Similar observations in high phenotypic diversity were reported by Akatwijuka et al. (2019); Apunyo et al. (2022), and Andiku et al. (2022). The observed high phenotypic diversity and moderate to high heritability for studied traits offer a higher selection response and confirm additive gene effects and the importance of these assembled lines (Enyew et al., 2022). Therefore, the assembled sorghum panel in Uganda is suitable for selection and crop improvement, offering implications for enhancing important traits and identifying key genes.

This study found significant positive correlations between plant height with panicle exsertion and grain yield, indicating that taller sorghum plants tend to have longer exsertion and produce higher yields consistent with findings by Akatwijuka et al. (2019), and Mohammed et al. (2015). This supports the possibility of simultaneous improvement of these traits through selection, making them effective indicators for high-yielding sorghum accessions (Andiku et al., 2022). A negative correlation between days to 50% flowering and grain yield observed in this study was also reported by previous studies by Akatwijuka et al. (2019) (*r* = -0.01), and Wanga et al. (2023) (*r* = -0.35). Although earliness is often associated with lower yields (X. Wang et al., 2020), this is often reversed when sorghum is grown under stress (Wanga et al., 2023), as evidenced by the inadequate rainfall received in 2021 and 2022 at NaSARRI (Katasi, 2021).

### 4.2 Genetic diversity and population structure

We examined the genetic diversity within the mini-core collection of 543 sorghum accessions assembled for breeding in Uganda originating from ICRISAT, NaSARRI, USA, ASERECA, and INTSORMIL using DArTSeq single nucleotide polymorphism (SNP) markers. In this study, we identified a total of 20,211 raw DArTSeq single nucleotide polymorphism (SNP) markers, called using sequencing data from 543 sampled sorghum accessions, which were narrowed to 7,156 high confidence SNP markers (35.4%) after filtering spread over the 10 chromosomes. An average marker density of 65.4 SNPs per Mb across the genome was observed over 543 accessions.

The PIC of the 7,156 SNPs ranged from 0.09 to 0.5, with an average polymorphism of 0.3. Our average PIC of 0.3 from the present study was slightly higher than those reported by Afolayan et al. (2019) (PIC = 0.24), Sejake et al. (2021) (PIC = 0.22), Enyew et al. (2022) (PIC = 0.24), and Yahaya et al. (2023) (PIC = 0.26) who also used SNPs to analyze sorghum germplasm collections. Therefore, our results showed that the SNP markers were informative, polymorphic, and sufficient to characterize the genetic diversity within the mini-core collection.

The average observed heterozygosity of 0.07 in this study aligns with findings from Enyew et al. (2022) (Ho = 0.06), who used SNP markers for sorghum analysis. However, our results had a lower Ho than previous studies conducted by Afolayan et al. (2019) (Ho = 0.22), and Yahaya et al. (2023) (Ho = 0.15) using SNP markers. The small Ho from our study was expected due to the inbreeding nature of sorghum, as it is a self-pollinating crop with a low outcrossing value (0-30%) (Paterson et al., 2004).

The gene diversity, also known as the expected heterozygosity (He), is a measure of genetic diversity within a population, indicating the proportion of heterozygotes under Hardy-Weingerg equilibrium ranged from 0.1 to 0.5 with a mean of 0.3, suggesting moderate levels of genetic diversity within the mini-core collection of sorghum accessions in Uganda. Our He results are consistent with Sejake et al. (2021) (He = 0.3) and Yahaya et al. (2023) (He = 0.32). Furthermore, this study reveals a disparity between average observed heterozygosity (0.07) and average expected heterozygosity (0.3), suggesting that inbreeding, genetic drift, or selection pressures could be reducing genetic variation (Mamo et al., 2023). Breeders should introduce diverse genetic material and promote outcrossing to increase genetic diversity and reduce inbreeding. Mbeyagala et al. (2012) reported similar findings of low genetic diversity using SSR markers and Enyew et al. (2022) using SNP markers. Enyew et al. (2022) and Yahaya et al. (2023) also reported consistent findings of low heterozygosity.

Population structure analysis is crucial for assessing genetic diversity and is an important step before conducting GWAS to uncover associations between markers and traits (Coates et al., 2009). In our study, both STRUCTURE results (optimal *K* = 2) and the PCA analyses indicated that the 543 *S. bicolor* accessions could be clustered into two sub-populations, with a few accessions (6.26%) with alleles inherited from both genetic subpopulations and the PCA results coincided with the STRUCTURE results. Furthermore, the dendrogram analysis (neighbor-joining tree) gave similar results. Overall, PCA and phylogenetic results agreed with results from the admixture-based model, indicating that the sorghum accessions were grouped into two groups. The grouping and pattern were dependent on the geographical origin of our study. Most accessions in both clusters originated from ICRISAT highlighting the importance of this Gene bank as a source and regional repository contributing to the genetic diversity of sorghum in African breeding programs (https://genebank.icrisat.org/). However, Motlhaodi et al. (2014) and Nemera et al. (2022) did not observe a clear grouping among Ethiopian accessions based on geographical origin.

The AMOVA revealed that the genetic variation among subpopulations (11.9%) was lower than that within subpopulations (88.1%). A comparable trend of higher genetic variation within subpopulations and lower genetic variation among subpopulations has been documented by Adugna (2014), using SSR markers; Nemera et al. (2022) using microsatellite markers; and Yahaya et al. (2023) using SNP markers. This pattern could be attributed to the self-pollinating nature of sorghum (Doggett, 1970; Dillon et al., 2007). This study also reported a low F_ST_ value (0.12) found between the two subpopulations, indicating a low genetic differentiation between the two subpopulations (Wright, 1965), consistent with findings in other studies (Enyew et al., 2021; Nemera et al., 2022; Yahaya et al., 2023). This suggests that the subpopulations are relatively similar genetically, which may limit the potential for breeding programs to develop new traits (Mamo et al., 2023). To address this, Ugandan breeders need to introduce new genetic material from more diverse sources to enhance genetic variation and improve the potential for developing desirable traits.

### 4.3 Linkage disequilibrium

Understanding linkage disequilibrium (LD) patterns in SNP markers is vital for genetic applications (Wang et al., 2013). LD informs study design in association studies to minimize false positives and enhance power thereby giving a more precise gene mapping (Morris et al., 2013; Shehzad and Okuno, 2020). Furthermore, in marker-assisted selection (MAS), LD helps to select individuals with desired traits, reducing time and costs in breeding (Thomson et al., 2010). This study assessed LD decay using 7156 SNP markers from 543 sorghum accessions. At the genome level average r^2^ was < 0.1. LD started at an r^2^ value of 0.45 and reached half-decay (r^2^ = 0.23) by 92.2kb. The observed decrease to r^2^ = 0.23 at 92.2 kb aligns with previous studies in sorghum, indicating an average LD decay rate between 15kb and 150kb (Morris et al., 2013; Kimani et al., 2020).

In contrast, to LD decay estimates of 15-20kb reported by Hamblin et al. (2005), our study observed higher LD decay estimates. However, our estimates were lower than LD decay estimates within 440-500kb reported by Marla et al. (2019) and Enyew et al. (2022). The difference in LD decay estimates may be attributed to the low genome coverage of markers from this study and the fact that sorghum is primarily a selfing species with occasional outcrossing which could lead to a higher LD compared to outcrossing species (Morris et al., 2013). The average r^2^ values across all sorghum chromosomes suggest a consistent rate of decay, from 0.06 to 0.07. This observation is slightly lower than reported LD decay rates in previous studies, which ranged from 0.09 to 0.12 (Wang et al., 2013; Enyew et al., 2022).

### 4.4 Genome-wide associations and candidate gene identifications for agronomic traits

Genome-wide association studies (GWAS) in sorghum have been instrumental in identifying novel marker-trait associations for major agronomic traits (Boyles et al., 2016; Zhao et al., 2016; Girma et al., 2019; Habyarimana et al., 2020). This study conducted in Uganda with a diverse panel of 543 sorghum breeding lines revealed significant insights into the genetic basis of key traits, paving the way for advancements in sorghum breeding practices. The research identified novel marker-trait associations (MTAs) and candidate genes, enhancing our understanding of sorghum’s genetic mechanisms, and providing a foundation for future research and application in crop improvement. In this study, there was sufficient statistical power in the GWAS using three-multi locus models (FarmCPU, MLM, and MLMM) to detect significant associations for yield-related traits (Yu et al., 2006; Segura et al., 2012; Liu et al., 2016).

#### 4.4.1 Plant height

Two significant MTAs, S2_16670214 and S2_5378133, located on chromosome 2, have been linked to plant height (PH) through the farmCPU model. The S2_5378133 variant is situated within the coding sequences of the candidate gene SORBI_3002G056000, spanning from position 5.376 to 5.386 Mbp, with a length of 70 kbp and encoding 1544 amino acids. This gene is known for its involvement in chromatin binding and exhibits expression in the whole stem during the seedling stage, as well as in the middle internode and the internode below the peduncle, according to previous studies by McCormick et al. (2018), and Makita et al. (2015). In addition, S2_16670214, also positioned on Chromosome 2 and associated with PH, shows aligns to SORBI_3002G124600 located between 16.665 and 16.674 Mbp, spanning a length of 2.7 to 8.1 kbp and encoding 800 amino acids. This gene is implicated in nucleic acid binding and demonstrates expression in the whole stem, as reported by Makita et al. (2015), and in the four last internodes at the top, according to Kebrom et al. (2017).

#### 4.4.2 Days to 50% flowering

Days to 50% flowering (DTF) in a genome-wide association study, two significant SNPs were identified to be associated with DTF. S5_3569592 on chromosome 5 is found within coding sequences of the candidate gene SORBI_3005G039000. This gene spans from 3.566 to 3.567 Mbp, with a length of 1.2 kbp, and consists of 278 amino acids and encodes for carbonate dehydratase activity and zinc ion binding and plays a crucial role in floral development, specifically in anther, pollen, spikelet, and lower panicle development (Davidson et al., 2012; Makita et al., 2015; Wang et al., 2018). Enyew et al. (2022), identified QTNs associated with days to 50% flowering on chromosome 5, including sbi982537 within the coding sequences of gene Sobic.001G230700 in Ethiopian sorghum landraces which encodes RING finger and E3 ubiquitin-protein ligase MIEL1, a homolog in rice involved in seedling development and flowering time regulation.

#### 4.4.3 Panicle exsertion

This study has identified three significant SNPs associated with panicle exsertion. The first SNP, S4_8921335, located on chromosome 4, is linked to the candidate gene SORBI_3004G099700. This gene spans from 8.916 to 8.920 Mbp, consists of 3.8 kbp, and encodes a protein with 1037 amino acids. SORBI_3004G099700 is involved in protein phosphorylation and is believed to play a role in regulating physiological processes. It is associated with protein binding and ATP binding functions and is linked to traits like flag leaf sheath development, the emergence of the first leaf, and the internode below the peduncle McCormick et al. (2018). The second SNP, S7_61349278, aligns with the gene SORBI_3007G180200 on chromosome 7. Measuring 2 kbp and extending from 61.366 to 61.370 Mbp, this gene encodes a protein comprising 300 amino acids. Although the exact function of this gene remains unknown, it is associated with early inflorescence and pistil development (Davidson et al., 2012), as well as growth in the higher internodes (McCormick et al., 2018). Lastly, the third SNP, S9_375005 on chromosome 9, aligns with the gene SORBI_3009G004200. It covers a length of 2.7 kbp and extends from 0.374 to 0.378 Mbp; this gene encodes a protein consisting of 607 amino acids. SORBI_3009G004200 plays a role in defense response and the regulation of the salicylic acid-mediated signaling pathway. It is associated with early inflorescence development and vegetative meristem function (Davidson et al., 2012), as well as panicle development and the internode below the peduncle (McCormick et al., 2018).

#### 4.4.4 Glume coverage

S4_56863263 on Chromosome 4 is associated with GC and aligns with the candidate gene SORBI_3004G219100. This gene spans from 56.862 to 56.865 Mbp, with a length of 3.2 kbp, and encodes for 302 amino acids. SORBI_3004G219100 is involved in the Glycosylphosphatidylinositol (GPI) anchor biosynthetic process in the cell membrane, which plays a role in pollen tube growth and germination. It is associated with emerging inflorescence and inflorescence development (Davidson et al., 2012).

#### 4.4.5 Grain yield

Grain yield (GY) is a crucial trait in cereal production, and its genetic basis has been the subject of extensive research in sorghum (Boyles et al., 2016; Enyew et al., 2022; Mulugeta et al., 2022). Two SNPs, S6_55680307 on chromosome 6 and S10_1446937 on chromosome 10, are linked to GY. Of these, S6_55680307 on chromosome 6 demonstrates a significantly higher effect size of 622.926 compared to S10_1446937 on chromosome 10, with an effect size of 312.385. These SNPs are within the coding sequences of candidate genes SORBI_3006G207100 and SORBI_3010G017700, respectively. The candidate gene SORBI_3006G207100 spans from 55.685 to 55.686 Mbp, with a length of 1.12 kbp encoding 74 amino acids. This gene is known to be involved in various plant tissues and developmental stages, including seed, seed 5 days after pollination, endosperm, inflorescence, and pericarp (Davidson et al., 2012). Furthermore, the candidate gene SORBI_3010G017700 is located between 1.447 and 1.449 Mbp, spanning 1.5 kbps and encoding 370 amino acids. This gene plays a role in pollen at the booting stage, spikelet development, and panicle formation (Makita et al., 2015; Wang et al., 2018).

## 5 Conclusions

This study addresses gaps in research within Uganda’s sorghum breeding program, employing SNP markers to assess genetic diversity and trait associations. The high phenotypic diversity among sorghum lines shows their potential for genetic studies and subsequent crop improvement strategies. However, disparities between observed and expected heterozygosity levels suggest the imperative need to implement strategies to enhance genetic diversity within the breeding populations. The identification of two genetic subpopulations with relatively low genetic differentiation emphasizes the importance of introducing diverse genetic materials to enrich breeding programs. Furthermore, the findings from genome-wide association studies offer valuable insights into the underlying genetic mechanisms governing key traits in sorghum. Looking ahead, it is essential to prioritize implementing strategies to enhance genetic diversity and continue leveraging genomic-assisted breeding approaches. Collaboration with international gene banks and research institutions can facilitate access to diverse germplasm, thus enriching the genetic pool for breeding programs. Furthermore, investing in capacity building and research infrastructure will support advanced genetic studies and enhance crop improvement efforts. Ultimately, sorghum breeding in Uganda stands to benefit significantly from harnessing SNP markers for genetic diversity assessment and trait association studies, thereby contributing to enhanced food security and livelihoods in the region. Breeders can accelerate breeding endeavors and genetic gain by fostering an improved understanding of sorghum genetics, genomics and collaborative efforts.

## Data Availability

The datasets presented in this study can be found in online repositories. The names of the repository/repositories and accession number(s) can be found in the article/**Supplementary Material**.
