# Supplementary material for: Genetic diversity, population structure, and a genome-wide association study of sorghum lines assembled for breeding in Uganda
Source: Front Plant Sci. 2024 Oct 7;15:1458179. doi: 10.3389/fpls.2024.1458179 (PMC11492802; doi:10.3389/fpls.2024.1458179)
Supplement: Supplementary file 1 [file DataSheet1.docx]

**Genetic diversity, population structure, and a genome-wide association study of sorghum lines assembled for breeding in Uganda**

Faizo Kasule^1,2,3^*****, Boris M.E Alladassi^2^, Charles John Aru^3^, Scovia Adikini^3^, Moses Biruma^3^, Michael Adrogu Ugen^3^, Ronald Kakeeto^3^, and Williams Esuma^3,4^

^1^Interdepartmental Genetics and Genomics (IGG), Iowa State University, Ames, IA 50011, USA

^2^Department of Agronomy, Iowa State University, Ames, IA 50011, USA

^3^National Semi-Arid Resources Research Institute (NaSARRI), P.O. Box 56, Soroti, Uganda

^4^National Crops Resources Research Institute (NaCRRI), Namulonge, P.O. Box 7084, Kampala, Uganda

*** Correspondence:**Faizo Kasule
[fkasule@iastate.edu](mailto:fkasule@iastate.edu)

Keywords: **DArT-Seq*,* genetic variation*,* GWAS*,* linkage disequilibrium*,* SNPs*,* sorghum**

**Supplementary tables**

**Table S1.** Passport data of the sorghum accessions used in this study

**(Excel file)**

**Table S2.** The descriptive statistics and genetic parameters of phenotypic traits

| Trait | Minimum | Maximum | Mean | V_G_ | V_GxE_ | V_e_ | *H^2^* |
| --- | --- | --- | --- | --- | --- | --- | --- |
| DTF | 58 | 117 | 83 | 16.6 | 0 | 28.8 | 0.54 |
| PH | 67 | 319.5 | 142.7 | 1058 | 118.8 | 115.2 | 0.9 |
| PE | 0 | 25.5 | 3 | 13.45 | 0 | 6.35 | 0.81 |
| GY | 140 | 8300 | 1100 | 93893.5 | 55333 | 144980 | 0.48 |

DTF = Days to 50% flowering (days), PH = Plant height (cm), PE = Panicle exsertion (cm), GY = Grain Yield (kg/ha), V_G_ = Genetic variance, V_GxE_ = Variance of genotype by environment effects; Ve = Error variance, *H^2^* = Broad-sense heritability

**Table S3.** A summary of the linkage disequilibrium (LD) analysis results among significant SNP marker pairs for each chromosome.

| Chr | Number of significant pairs (*p* <= 0.01) | Average *r*2 | Median *r*2 | Number of pairs in complete LD (*r*2 = 1) |
| --- | --- | --- | --- | --- |
| 1 | 254,769 | 0.068 | 0.039 | 980 |
| 2 | 235,282 | 0.068 | 0.038 | 963 |
| 3 | 217,804 | 0.069 | 0.040 | 925 |
| 4 | 113,678 | 0.072 | 0.039 | 650 |
| 5 | 100,121 | 0.075 | 0.041 | 620 |
| 6 | 148,275 | 0.067 | 0.038 | 761 |
| 7 | 68,482 | 0.069 | 0.039 | 500 |
| 8 | 75,175 | 0.077 | 0.044 | 516 |
| 9 | 97,920 | 0.071 | 0.040 | 607 |
| 10 | 106,725 | 0.076 | 0.042 | 628 |
| Whole genome | 1,418,231 | 0.070 | 0.040 | 7,150 |

Chr = chromosome

**Table S4**. List of significant quantitative trait nucleotides (QTNs) identified by three multi-locus genome-wide association study (GWAS) methods for sorghum quantitative traits.

| Trait | Env | SNP | Chr | Position (bp) | *p*.value | *q.*value | MAF | Effect | Model |
| --- | --- | --- | --- | --- | --- | --- | --- | --- | --- |
| PH | 2021 | S2_16670214 | 2 | 16670214 | 2.00E-06 | 0.02 | 0.11 | 12.27 | FarmCPU |
|  | Across | S2_5378133 | 2 | 5378133 | 1.00E-06 | 0.01 | 0.37 | -8.43 | FarmCPU |
| DTF | 2021 | S3_61344759 | 3 | 61344759 | 5.00E-06 | 0.03 | 0.25 | 2.22 | FarmCPU |
|  |  |  |  |  | 7.00E-06 | 0.05 | 0.25 | 2.23 | MLM |
|  |  |  |  |  | 5.00E-06 | 0.04 | 0.25 | 2.23 | MLMM |
|  | Across | S3_61344759 | 3 | 61344759 | 4.00E-08 | 0 | 0.25 | 2.43 | FarmCPU |
|  |  |  |  |  | 9.00E-06 | 0.06 | 0.25 | 2.25 | MLM |
|  |  |  |  |  | 3.00E-08 | 0 | 0.25 | 2.36 | MLMM |
|  |  | S5_3569592 | 5 | 3569592 | 2.00E-06 | 0.01 | 0.09 | -2.5 | MLMM |
| PE | 2022 | S4_8921335 | 4 | 8921335 | 3.00E-06 | 0.01 | 0.43 | -1.04 | FarmCPU |
|  |  | S5_60770709 | 5 | 60770709 | 5.00E-08 | 0 | 0.26 | 1.67 | FarmCPU |
|  | Across | S7_61349278 | 7 | 61349278 | 3.00E-09 | 0 | 0.09 | 1.95 | FarmCPU |
|  |  | S9_375005 | 9 | 375005 | 7.00E-06 | 0.03 | 0.09 | -1.22 | FarmCPU |
| GC | 2021 | S4_56863263 | 4 | 56863263 | 5.00E-07 | 0 | 0.05 | 0.18 | MLMM |
| GY | 2022 | S2_4351947 | 2 | 4351947 | 3.00E-08 | 0 | 0.5 | -129.49 | FarmCPU |
|  |  |  |  |  | 8.00E-06 | 0.02 | 0.5 | -191.16 | MLM |
|  |  | S6_55680307 | 6 | 55680307 | 4.00E-08 | 0 | 0.06 | 421.76 | FarmCPU |
|  |  |  |  |  | 8.00E-08 | 0 | 0.06 | 622.93 | MLM |
|  |  |  |  |  | 1.00E-08 | 0 | 0.06 | 592.64 | MLMM |
|  |  | S10_1446937 | 10 | 1446937 | 8.00E-08 | 0 | 0.05 | 259.94 | FarmCPU |
|  |  |  |  |  | 9.00E-06 | 0.02 | 0.05 | 312.38 | MLM |
|  |  |  |  |  | 2.00E-06 | 0.01 | 0.05 | 305.83 | MLMM |

PH = plant height, DTF = days to 50% flowering, PE = panicle exsertion, GC = glume coverage, GY = grain yield, Env = environment, MAF = minor allele frequency, FarmCPU = Fixed and random model circulating probability unification, MLM = Mixed Linear Model, MLMM = Multiple Loci Linear Mixed Model.

**Supplementary figures**

**Supplementary Figure S1:** Distribution of SNPs within the 1Mb window size across the 10 chromosomes of sorghum (Marker density unfiltered)

**Supplementary Figure S2**: A scatter plot of the 543 sorghum accessions showing their distribution along PC1 and PC2, inferring the membership of the accessions based on country of source (A) and origin (B).

**Supplementary Figure S3:** The scatter plot of genome-wide linkage disequilibrium (LD) decay was determined based on the r^2^ values of the marker pairs. Different colors indicate the different chromosomes.
