# Supplementary figures and images for: Genetic diversity, population structure, and a genome-wide association study of sorghum lines assembled for breeding in Uganda
Source: Front Plant Sci. 2024 Oct 7;15:1458179. doi: 10.3389/fpls.2024.1458179 (PMC11492802; doi:10.3389/fpls.2024.1458179)

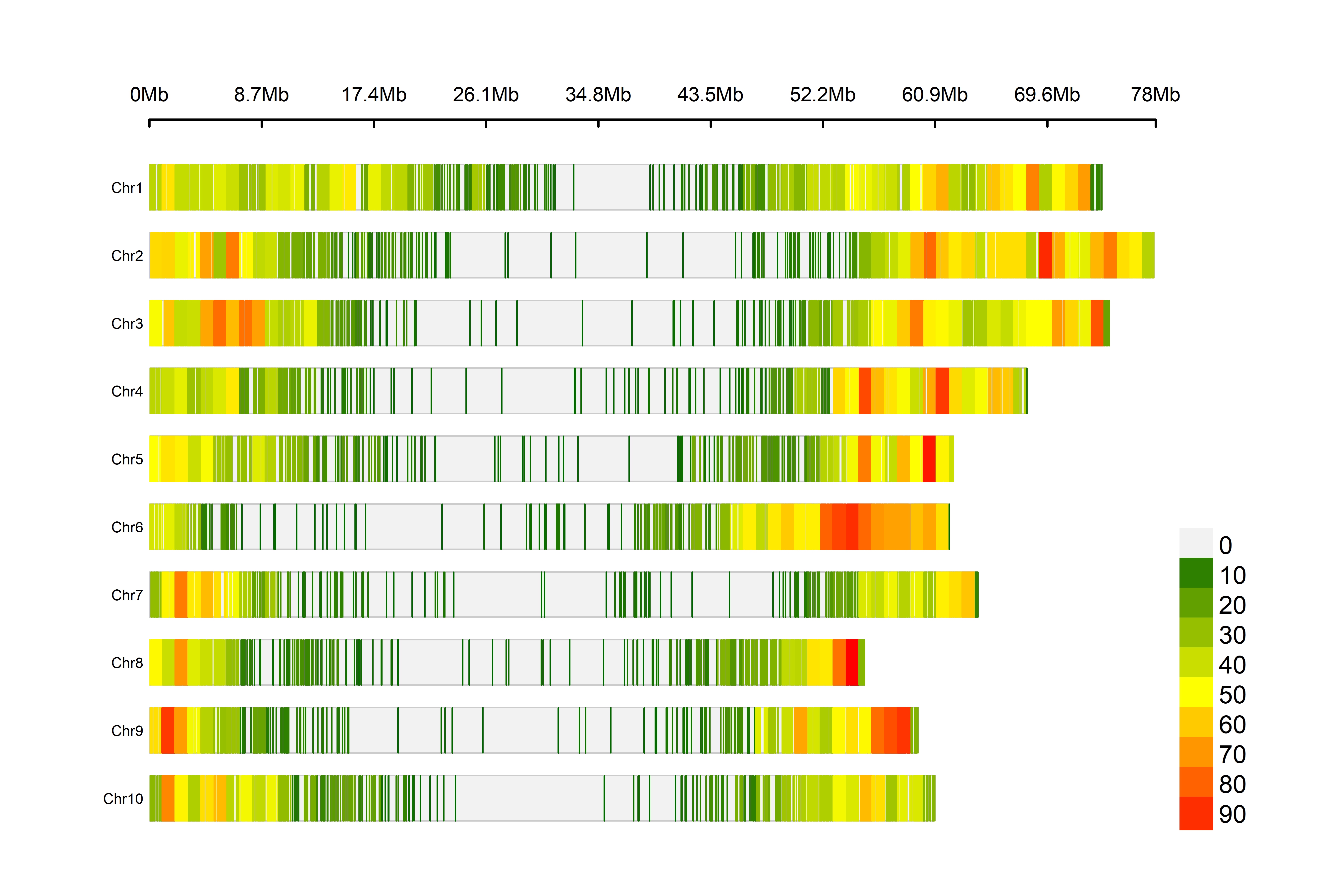

Supplement: Supplementary file 2 [file Image1.jpeg]

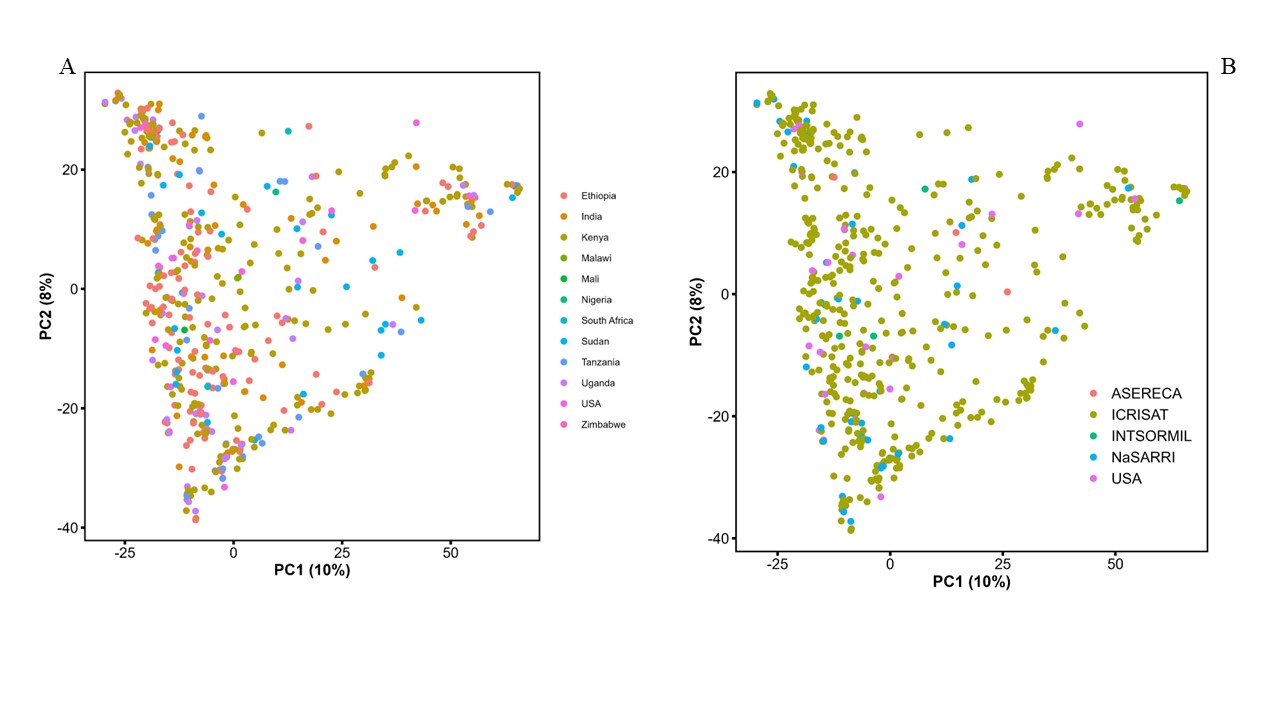

Supplement: Supplementary file 3 [file Image2.jpeg]

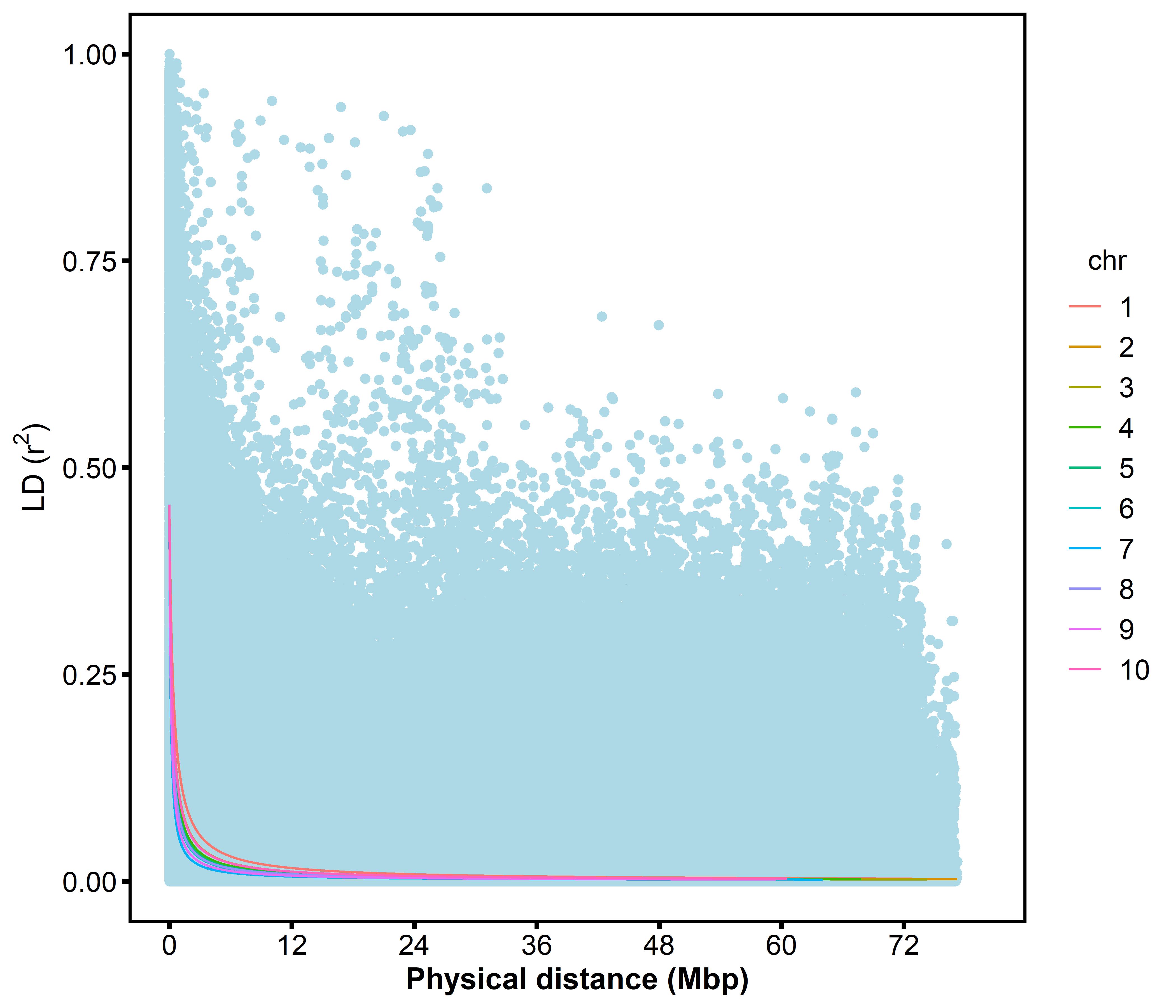

Supplement: Supplementary file 4 [file Image3.jpeg]
